# Enhancing the comfort of hospitalized elderly patients: pain management strategies for painful nursing procedures

**DOI:** 10.3389/fmed.2024.1390695

**Published:** 2024-06-19

**Authors:** Camilla Elena Magi, Yari Longobucco, Carla Amato, Claudia Camedda, Chiara Balestri, Khadija El Aoufy, Paolo Iovino, Stefano Bambi, Laura Rasero

**Affiliations:** ^1^Department of Health Sciences, University of Florence, Florence, Italy; ^2^Department of Medical and Surgical Sciences, University of Bologna, Bologna, Italy; ^3^Department of Head and Neck District Diseases, Istituto di Ricovero e Cura a Carattere Scientifico (IRCCS) Azienda Ospedaliero-Universitaria di Bologna, Bologna, Italy

**Keywords:** pain, nursing, elderly, painful procedures, advocacy, nursing approach, non-pharmacological intervention, pain management

## Introduction

One of the main nursing responsibilities is to ensure patient wellbeing and comfort. In particular, comfort is closely related to nursing practices (1). Comfort is defined as relief from pain, and emotional and physical distress (2). However, hospitalized elderly patients often undergo several nursing procedures interventions during hospitalization, which are crucial for addressing their complex health needs (3). This extensive attention often exerts a toll, primarily in the form of pain (3). Effectively managing pain in this demographic group extends beyond clinical considerations because it represents a profound moral and ethical imperative given the distinctive challenges encountered by elderly individuals in hospital settings (4). These challenges include a higher prevalence of acute and chronic diseases, age-related physiological alterations, and cognitive impairment or delirium, which can seriously affect pain management (5).

Pain perception represents an intrinsic facet of human experience, which defies simplification as it is a profound subjective phenomenon shaped by an array of determinants (6). Psychological, physiological, and sociocultural factors contribute significantly to shaping how individuals perceive and cope with pain (7, 8). For elderly patients whose experiences of pain are often compounded by these multifarious factors, a tailored and specialized approach to pain management is essential.

During their hospital stay, elderly individuals undergo nursing procedures aimed at addressing their clinical condition (9). Nursing procedures encompass both basic (e.g., fundamentals of care) and advanced activities. The most frequently painful procedures in older adults include mobilization, wound dressing, bladder catheterization, and needle-related procedures, which are emerging as the most common sources of pain among elderly patients. These indispensable procedures, which are crucial during hospitalization, frequently induce discomfort (10). Unfortunately, inadequate pain management frequently persists, leading to heightened anticipatory responses to pain stimuli during subsequent procedures (11). This aspect has been widely explored in different types of patients, such as pediatric (12, 13), but evidence in elderly patients is still lacking.

From this perspective, recognizing the pivotal importance of the comfort of elderly patients and the inherently subjective nature of pain perception, it is imperative to identify effective pain management strategies customized to the unique needs and preferences of each individual patient (14). Such strategies should encompass a comprehensive spectrum spanning both pharmacological and non-pharmacological interventions (15). By addressing these pain experiences more frequently and empirically, healthcare providers can significantly improve the overall quality of life of elderly patients during their hospitalization (16).

While the comfort of older adults during hospitalization is a key concept, there is limited literature and insufficient evidence to indicate the optimal strategies for each individual nursing procedure that may cause discomfort. Furthermore, this issue is underemphasized in clinical practice, often relegated to a secondary consideration at the expense of providing quality, patient-centered care tailored to the specific needs of each hospitalized patient.

The aim of this article is to delve into the critical significance of managing acute procedure-related pain, presenting a compendium of efficacious strategies aimed at bridging the existing gap in pain management in hospitalized elderly individuals. From this perspective, this paper examines the challenges related to pain management in this specific population group, presenting evidence-based approaches that can be easily integrated among the acts of care by nurses, in order to enhance the quality of care provided to elderly patients during their hospital stay.

## Strategies for enhancing pain management

Effective management of pain during routine nursing procedures, such as blood draws, peripheral venous catheter placement, injections, urinary catheterization, tube insertions, and wound dressing changes, is essential for optimizing patient comfort and overall quality of healthcare delivery (3, 17).

The literature provides valuable insights into various strategies adopted by nurses, including non-pharmacological and technological approaches, to enhance patient experiences and minimize pain perception (18, 19).

**Figure 1:**
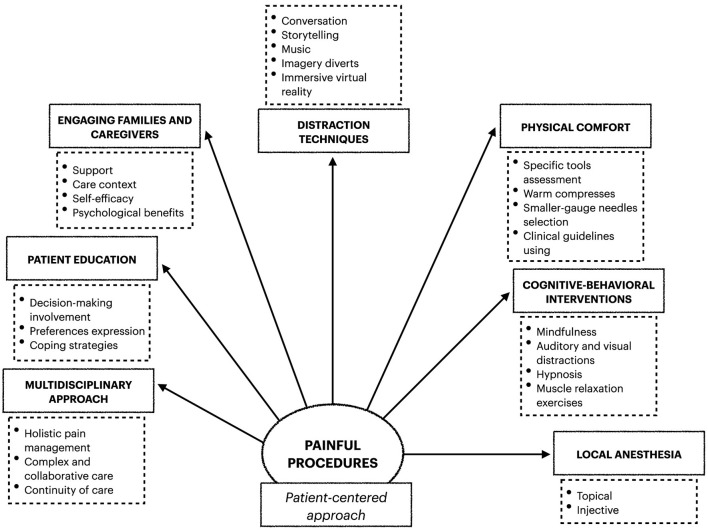
Pain management strategies.

These strategies encompass the nuances of evidence-based approaches, with different levels of invasiveness for elderly patients, as shown in Figure 1.

### Multidisciplinary approach

First, a collaborative approach among healthcare professionals, including nurses, physicians, and pain specialists, is essential for the development of standardized pain management protocols. This interdisciplinary approach ensures that the most appropriate and effective pain control strategies are consistently employed, reducing painful procedures, such as through the implementation of mid- or long-term devices (e.g., peripherally inserted central catheters) (20).

Furthermore, embracing the principles of “complex and collaborative care” (21) signifies a shift in mindset and paradigms toward recognizing the multidimensional nature of patient needs. It involves combining diverse perspectives and valuing various resources and their composition (21). This requires valuing the active involvement of the patient and fostering new opportunities for inter-professional collaboration. This approach entails cooperation among various care resources and a reimagined conception and organization of the continuum of care, ensuring a holistic and integrated approach to the comfort of elderly patients (21).

### Patient education

Educating patients about the procedure, its purpose, and potential sensations can significantly contribute to reducing pain perception (22). In fact, patient involvement in decision-making and providing them with the opportunity to express their preferences can empower individuals and enhance their sense of control during procedures (23). Education can reduce anticipatory pain (24), the modulation of which can reduce anxiety and, consequently, pain perception, and promote positive and effective coping strategies (24, 25). Nevertheless, the patient education and engagement process is strictly related to patient satisfaction with the hospitalization experience, reducing healthcare avoidance (26).

### Engaging families and caregivers

Actively engaging families and caregivers in the pain management process can significantly reduce patient anxiety, enhance overall support, and provide a more empathetic and comprehensive care environment. The involvement of families and caregivers can make a substantial contribution to the patient's comfort and, consequently, pain management (27) in order to improve the effects of the chosen interventions (28). Furthermore, in elderly patients with dementia, caregivers can play a pivotal role in the surveillance of pain levels (29), although there is evidence that self-efficacy support interventions need to be planned to support this role (30). Nevertheless, caregivers' engagement also shows benefits such as anxiety, stress, and depression (31).

### Distraction techniques

Distraction techniques have proven to be valuable in reducing pain perception during procedures (32, 33). Engaging patients in calming conversations, storytelling, soothing music, and guided imagery diverts their attention from the procedure itself. These techniques have successfully mitigated subjective experiences of pain (34). Furthermore, the integration of immersive virtual reality (VR) technology into healthcare settings offers an innovative means of transporting patients to alternative environments, effectively diminishing pain perception during procedures (35). However, although VR is a well-known, useful, and scalable intervention, the costs of this technology still represent an implementation barrier (21), and cognitive impairment can hinder its application depending on the level of severity (36).

### Physical comfort

Physical comfort plays a vital role in pain management, particularly during nursing procedures. In this demographic group, optimizing physical comfort is essential for controlling the symptoms and ensuring patient comfort. personalized care should adopt a preventive approach based on evaluation through specific assessment tools. For example, as reported in the literature, over a third of adults exhibit difficult venous access (DiVA) (37), making the evaluation of venous heritage essential. From this perspective, adopting rating scales, such as the ADIVA scale, makes it possible to identify at-risk patients and adopt personalized strategies (38).

Utilizing warm compresses to facilitate vein dilation is instrumental in enhancing patient comfort and reducing procedural discomfort (39). Additionally, careful selection of smaller-gauge needles is imperative to minimize tissue trauma and discomfort during venipuncture (40), aligning with best practices outlined in clinical guidelines (41). Moreover, employing appropriate techniques and adopting a gentle approach in handling elderly patients during procedures are integral components of pain management strategies, all contributing to minimizing discomfort (40).

### Cognitive-behavioral interventions

Cognitive-behavioral interventions include mindfulness meditation, auditory and visual distractions, hypnosis, and progressive muscle relaxation exercises (42). These interventions offer psychological tools for patients to effectively modulate pain perception and emotional responses (15, 43). Hypnosis is an effective intervention in pain management (44), particularly in wound dressing (45). In patients without cognitive impairment, hypnotic susceptibility can be assessed with easy-to-administer tools such as the Stanford Hypnotic Susceptibility Scale (46).

### Pharmacological treatment

Nursing procedures that require localized pain relief should be treated pharmacologically with options such as local anesthesia and systemic analgesics.

Local anesthesia, whether administered topically or via injections, has shown significant efficacy, particularly in procedures where localized pain management is pivotal for patient comfort and procedural success (47–49). When local anesthesia is applied in a specific area, immediate relief is provided, allowing procedures to be performed with minimal discomfort to the patient. Nevertheless, analgesics are tailored to individual patient needs (47), considering factors such as medical history, and concurrent medications, to optimize pain management outcomes and minimize potential adverse effects (50). However, in order to promote a safe and comfortable procedural experience for elderly patients, other approaches besides local anesthesia should be considered, especially for lengthy procedures. For example, following a multidisciplinary approach, a timely adoption of opioids or other systemic analgesia techniques may be adopted for painful wound-related procedures (51).

## Discussion

In the context of pain management during nursing procedures, there are a plethora of techniques and strategies to reduce discomfort in elderly patients (Figure 1). These techniques and strategies encompass different approaches that fit tailored and personalized care. The key concept of pain management emphasizes the importance of a patient-centered approach, highlighting the multitude of factors that influence treatment decisions within each individual's unique clinical and social context (52). This involves meticulous evaluation of pain levels, consideration of medical history, and recognition of individual needs and preferences (53). In this landscape, it is crucial to acknowledge the subjective nature of pain perception and to recognize other contributing factors, such as anxiety, which can amplify pain experience (52). In this perspective, the inclusion of non-pharmacological strategies in the nursing care plan, such as distraction techniques, is advisable. These dynamic strategies are continually evolving, alongside technological advancements and ongoing research. The overarching goal remains consistent with alleviating pain and enhancing the overall comfort of elderly patients. Healthcare providers can deliver high-quality care while prioritizing patient comfort by effectively managing acute pain and minimizing distress during procedures.

However, the existing literature and evidence concerning this topic are still lacking, underscoring the need for further exploration. Older adults are exposed to altered peripheral nerve conductivity, leading to a higher likelihood of developing pain (54). Moreover, aging is characterized by a low-grade inflammatory condition, known as “inflammation,” which affect the nociceptive system (55). Furthermore, the pharmacodynamics of analgesics are altered in older people as age-related changes in pain processing (56), enhancing the importance of non-pharmacological approaches.

Therefore, in general, managing pain related to nursing procedures involves integrating technical-operational skills with relational-communication abilities. Nevertheless, one of the key nursing interventions is advocacy, which implies both ethical and clinical relevance in nursing practice (57). Advocacy can enhance the overall quality of life of elderly patients by effectively documenting and reporting procedural pain, evaluating pain care skills, and identifying educational needs among the working group (57). We should not forget that pain management in elderly people who are unable to verbalize must be oriented to interventions that prevent the experience of pain before the beginning of a medical or nursing procedure (56).

This paper aims to represent a call to action at all levels, from the patient to healthcare management, through caregivers and individual healthcare professionals, advocating for an improvement in the quality of the evidence available on this increasingly discussed topic. By fostering a deeper understanding of pain management strategies and their impact on patient outcomes, we can collectively contribute to advancements in nursing practices and enhance the quality of care delivered to patients.

## Author contributions

CM: Conceptualization, Methodology, Project administration, Visualization, Writing – original draft, Writing – review & editing. YL: Conceptualization, Methodology, Visualization, Writing – original draft, Writing – review & editing. CA: Visualization, Writing – review & editing. CC: Writing – review & editing. CB: Writing – review & editing. KE: Writing – review & editing. PI: Writing – review & editing. SB: Methodology, Supervision, Writing – review & editing. LR: Conceptualization, Methodology, Project administration, Supervision, Writing – review & editing.
